# The Patient‐Centred Interdental Cleaning Concept—Consensus Based on a Round Table

**DOI:** 10.1111/idh.70009

**Published:** 2026-01-26

**Authors:** Tim M. J. A. Thomassen, Dagmar E. Slot, Christian Graetz, Nicola Discepoli, Johannes C. Ehrenthal, Cornelia Frese, Inmaculada Tomás, Christof E. Dörfer, Fridus (G. A.) Van der Weijden, Sonja Sälzer

**Affiliations:** ^1^ Department of Periodontology Academic Centre for Dentistry Amsterdam (ACTA) University of Amsterdam and Vrije Universiteit Amsterdam Amsterdam the Netherlands; ^2^ Clinic for Conservative Dentistry and Periodontology School for Dental Medicine, Christian‐Albrechts‐University Kiel Kiel Germany; ^3^ Unit of Periodontology, Department of Medical Biotechnologies University of Siena Siena Italy; ^4^ Department of Psychology University of Cologne Cologne Germany; ^5^ Department of Conservative Dentistry, Clinic for Oral, Dental and Maxillofacial Diseases, Dental School University Hospital Heidelberg, Heidelberg University Heidelberg Germany; ^6^ Oral Sciences Research Group, Special Needs Unit, Department of Surgery and Medical‐Surgical Specialties, School of Medicine and Dentistry Universidade de Santiago de Compostela, Health Research Institute of Santiago (IDIS) Santiago Spain

**Keywords:** bleeding, dental care professional, gingivitis, interdental brushes, interdental cleaning, periodontal disease, plaque

## Abstract

**Aim:**

To consolidate clinical and scientific evidence and develop personalised recommendations for the optimal use of interdental devices, addressing the diverse needs of patients with varying oral health conditions based on the outcomes of an expert meeting.

**Material and Methods:**

A round table meeting was convened, gathering nine qualified experts from various European countries, including clinicians and researchers*.* Through tailored pre‐formulated questions and facilitated group discussions, well‐defined clinical recommendations and considerations for future research were developed, addressing general guidelines and specific interdental hygiene recommendations alongside potential barriers to implementation.

**Results:**

General advice emphasises toothbrushing with fluoride toothpaste and integrating interdental cleaning into daily routines. Depending on early clinical signs and symptoms of caries and periodontal disease, interdental cleaning should become a consistent part of daily care. In general, interdental brushes are the preferred option. However, specific recommendations address conditions such as gingivitis, periodontitis, dental implants, caries, removable prostheses and orthodontic treatments. Recommendations also highlight various age groups, emphasising personalised approaches and prioritising interdental cleaning based on clinical indicators. In addition to the clinical situation, individual preferences and dexterity should be considered. For example, non‐wired interdental cleaning devices may be more suitable for novice users, while an oral irrigator may be recommended for those with limited dexterity—for example, older adults or patients with special needs.

**Discussion and Conclusion:**

Barriers to implementing interdental cleaning recommendations must be explored to provide practical solutions for the general population and dental care professionals. Strategies should include developing easily translatable educational content endorsed by professional societies to raise awareness, promoting behaviour change, tailoring care to individual needs, improving product usability, managing costs and addressing discomfort. It is also essential to align recommendations within the dental profession and prioritise interdental care in clinical practice, emphasising evidence‐based guidelines and collaborative education efforts to ensure effective interdental hygiene practices are adopted.

## 1 Introduction

Periodontal diseases and dental caries are highly prevalent conditions in humans and are the leading causes of tooth loss. These issues can affect food intake, potentially leading to adverse nutritional consequences, and can negatively impact self‐esteem and overall quality of life. Both conditions have mutual risk factors, including the presence of a biofilm (dental plaque) [1].

Caries, or tooth decay, occurs when there is an imbalance in the ongoing cycle of demineralisation and remineralisation in tooth enamel. This situation occurs when organic acids produced by cariogenic bacteria in dental plaque interact with fermentable carbohydrates (primarily sugars), leading to demineralisation [2]. The buildup of a biofilm both at and beneath the gingival margin, within which dysbiosis develops, is the primary risk factor for periodontal diseases, including gingivitis and periodontitis as well as peri‐implantitis. The condition is associated with an inappropriate and destructive host inflammatory immune response. Management of gingivitis is a primary prevention strategy for periodontitis and a secondary prevention strategy for recurrent periodontitis [3]. Therefore, effectively managing and removing plaque is fundamental in the prevention of caries and periodontal diseases [2, 3].

Various methods are available for mechanically removing dental plaque from teeth, with toothbrushes generally regarded as the most effective option [4, 5, 6]. In recent decades, several studies have tested and compared the efficacy of manual toothbrushes (MTBs) and powered toothbrushes (PTBs). Generally, research has found that PTBs are more effective than MTBs in reducing dental plaque, gingivitis and bleeding [7, 8, 9, 10, 11]. While a toothbrush effectively removes plaque from buccal, lingual and occlusal surfaces, it does not thoroughly clean the interdental area if biofilm growth is present [3]. Although, it is not clear how much biofilm has to be removed to attain symbiotic conditions, according to the actual knowledge an optimal interdental cleaning is essential for preserving gingival health, especially for secondary prevention. To effective proper interdental hygiene, it is essential to use a device that can effectively reach between adjacent teeth while also being convenient for the user [12]. This can be accomplished using various tools such as interdental brushes (IDB), non‐wire interdental cleaning devices (NWICD), floss, wood sticks and oral irrigators (OI). If interdental spaces are open, it is evident that thorough interdental cleaning is an indispensable component of oral self‐care [13].

In the last two decades, several systematic reviews on interdental cleaning have been published. The earliest review on IDBs concluded that they provide better plaque removal than brushing alone when used alongside toothbrushing [14]. In addition, their included studies showed a positive significant difference using IDBs with respect to the bleeding scores and probing pocket depth [14].

A Cochrane review found only one study assessing the effectiveness of toothbrushing combined with interdental brushing compared to toothbrushing alone. The evidence supporting a reduction in gingivitis and plaque after 1 month was insubstantial [15]. A Cochrane revision on all interdental cleaning devices concluded that using floss or interdental brushes in addition to toothbrushing may reduce gingivitis or plaque, or both, more than toothbrushing alone. Interdental brushes may be more effective than floss [16]. A meta‐review (an umbrella or systematic review) of systematic reviews regarding interdental cleaning concluded that IDBs are the most effective devices for removing interdental plaque and are most appreciated by patients. In the meta‐review, most of the available studies found that flossing was generally ineffective for plaque removal. However, all the investigated devices for interdental self‐care seemed to support the management of gingivitis to a varying extent [13]. One systematic review and meta‐analysis focusing on self‐care of periodontal maintenance patients concluded that no definitive ‘evidence‐based’ recommendation could be made for specific oral hygiene devices. This uncertainty arises from the limited number of included studies and the low certainty of evidence [17].

These findings were supported by a less rigid review and affirm that interdental brushes confer a significant advantage over toothbrushing only, especially for periodontal patients with open interdental areas [18]. In addition, the authors noted that no one‐size‐fits‐all solution exists for interdental cleaning aids and that the choice of an effective interdental cleaning tool is contingent on factors such as ease of use, size of interdental spaces, user acceptance, manual dexterity and individual motivation.

NWICDs, made of rubber or silicone, are a recent development and could be a viable alternative to conventional interdental brushes. They have proved effective in plaque removal and gingivitis reduction, with further benefits of greater patient compliance and acceptance in terms of comfort and willingness to buy the product [19]. However, the current body of evidence supporting their efficacy lacks substantiation [18, 19].

Since the early 1960s, OI devices for home use have been the subject of extensive research in both laboratory and clinical environments [20, 21, 22]. These devices generally function by utilising a pulsating stream of water to remove plaque and soft debris from teeth. OIs come in a variety of designs, each offering unique features and functionalities. Some models include adjustable pressure settings, specialised tips and built‐in timers, while others may additional microbubbles in the water flow to improve cleaning effectiveness [23, 24, 25]. A study from less than a decade ago, using Bayesian network meta‐analysis to compare the efficacy of 10 interdental oral hygiene aids, focused on their impact on gingival inflammation. IDBs and OI were the most effective in reducing the gingival index, significantly outperforming other aids like toothpicks and floss, which ranked lowest. While the findings offer a global efficacy ranking, the actual patient‐perceived benefits remain uncertain due to the reliance on clinical rather than subjective outcomes [26].

A recent literature review presented an evidence‐based decision‐making tree that advocates for personalised approaches to interdental cleaning [27]. This approach considers factors like embrasure size and individual‐specific conditions such as manual dexterity and motivation. The basis of this decision‐making was site‐specific depending on whether the type of interdental space is closed or open. From a consumer perspective, individual characteristics, such as age and dental considerations (e.g., removable prostheses, orthodontic appliances or dental implants), are more relevant and easier to understand. However, aspects of acceptability and ease of use are not given adequate consideration. Furthermore, the full text of the paper with the decision‐making tree is not easily accessible. By presenting research findings through open access, clinicians can access the most recent and pertinent studies available. This availability reinforces the foundation of evidence‐based practices and enhances clinical decision‐making, ultimately benefiting patient care.

It is the role of dental care professionals to provide appropriate oral hygiene advice to patients founded on current and reliable evidence. To facilitate this process, an expert meeting was convened to consolidate the currently available clinical evidence and formulate recommendations based on individual characteristics for the practical use of interdental devices in healthy and diseased mineralised tissues (e.g., proximal caries lesions) and soft tissues (e.g., gingivitis, periodontitis and peri‐implant disease) [28].

## 2 Methods

A round table meeting was initiated by four dental care professionals (GAW, CD, CG and DES) to evaluate clinical evidence regarding the use of interdental cleaning devices and develop practical recommendations for daily dental care. To uphold standards of transparency and rigour in the research methodology, the new Accurate Consensus Reporting Document (ACCORD) guideline was implemented for consensus methods in biomedicine [29], when applicable. The international meeting took place from October 28 to 30, 2023, in Barcelona. Nine experts, comprising clinicians and/or researchers from diverse European countries, participated. Within this group, two individuals (TT and SS) were tasked with documenting group discussions and consolidating the final statements. The remaining seven were given tailored, pre‐formulated questions and tasked with preparing a 30‐min presentation centred around their designated questions to serve as the initial step of the discussion (see Appendix S1). The pre‐defined questions, established by the initiators following a comprehensive group discussion, focused on three themes: research methodology, clinical application and specific patient groups. The questions were designed to facilitate a deeper understanding of the existing evidence. To establish a solid, evidence‐based foundation, the experts systematically reviewed the literature for each question, carefully evaluating the relevant studies. This process aimed to support a well‐organised and methodologically thorough exchange during the group discussion chaired by CD. The relevant scientific support was shared in advance via a digital platform. As part of the evidence synthesis, the group also developed a comparative ranking of interdental cleaning devices, based on the strength and consistency of the available data. During the round table discussion, each participant had an opportunity to contribute and influence the conversation, maintaining a central focus. The statements were collected on the digital platform, allowing the group to fine‐tune the wording and propose additions. These statements were then presented centrally and finalised by expert consensus. This process was followed by the development of clinical recommendations and considerations for future research.

The recommendations that emerged from this roundtable are presented in two stages: general guidelines for interdental cleaning, covering aspects such as the rationale, target population, effective techniques and tools; and specific interdental oral hygiene recommendations concentrating on various factors, including conditions, life stages, and dental prosthetics and devices. The discussion section of this paper addresses potential barriers to implementing the recommendations, describing obstacles and corresponding solutions. Table 1 provides a visual summary of these recommendations, consolidating the available literature and the expert opinions, resulting in the key points from the roundtable discussions.

**TABLE 1:** Recommendations for interdental cleaning device utilisation across different patient groups.

Devices^	IDB	Floss	Non‐wire ICD	Wood sticks	Oral irrigator
Adults & adolescents					
Healthy	<‐>	<‐>	<‐>	<‐>	<‐>
Proximal caries lesions/secondary lesions in need of control**	1st	*	Novice	Novice	2nd
Gingivitis	1st **	*	Novice	Novice	2nd
Periodontitis/reduced periodontium/proximal root caries	1st	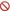	Novice	Novice	2nd
Peri‐implant mucositis	1st	*§	Novice	Novice	2nd
Peri‐implantitis	1st	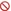 §	Novice	Novice	2nd
Dental implants	1st	*§	Novice	n.E.	2nd
Removable prosthesis	1st	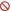	Novice	n.E.	n.E.
Patients with fixed orthodontic appliances (between wire &tooth surface)	1st	n.E.	Novice	n.E.	2nd
Children					
Healthy	<−>	<−>	<−>	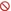	<−>
With clinical signs and symptoms	Hypomineralisation	1st	Novice	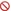	n.E.
Special needs patients/older people					
	1st	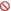	Novice	Novice	Inadequate dexterity

*Note:* 1st Primarily recommended. 2nd Secondarily recommended. 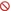 not recommended in general, exceptions only. <‐> Interdental cleaning devices are not needed in general, but on an individual basis only. *Healthy*: intact papilla/without signs of periodontal inflammation or caries lesions. ^For detailed instruction per devices, see Online Appendix. *When IDB will not pass through the interproximal area without trauma, floss may be used. **In case of proximal caries lesions or secondary lesions use the interdental device in combination with a non‐abrasive fluoride gel. ^§^Use dental floss for implants when the interdental space or contour of the supra‐structure does not allow for IDB. Warning: Floss should not to be used, if rough surface parts are likely to be exposed! *Novice*: For novice ID cleaners. *n.E*.: no sufficient evidence.

## 3 Results

### 3.1 Research Methodology

#### 3.1.1 What Is the Recommended Methodology for Investigating the Efficacy of Interdental Cleaning Devices?

Both in vitro and in vivo research focus on surrogate parameters that assess efficacy and safety. In vitro studies predominantly analyse mechanical variables, such as surface contact and insertion force, using models of varying sophistication [30]. In vivo studies often consider the reduction in interdental bleeding over time as the most reliable surrogate for changes in the level of interdental inflammation [31]. Assessing plaque reduction, which is a direct outcome of mechanical cleaning, can only be simulated in vitro using artificial plaque. In the clinical setting, it is challenging to verify plaque reduction for interdental surfaces between adjacent teeth and even impossible in the case of filled interdental spaces. Alternative methods, such as simulating interdental biofilms, may not be sufficiently validated or practical for current clinical studies.

#### 3.1.2 How Can Standardisation of the Choice of Interdental Brush in Dental Practice Be Facilitated?

ISO 16409:2016 [32] specifies requirements, test methods and size classification for the qualitative evaluation of manual interdental brushes with a round cross‐section of the brush head and consisting of a wired stem with inserted filaments. The standard also specifies the accompanying information, including manufacturer's instructions for use and label packaging. The passage hole diameter (PHD) of interdental brushes from various manufacturers ranges from 0.6 mm to 5.2 mm. Around 90% of these brushes have a PHD of ≤ 2.0 mm [33, 34]. The current uneven distribution of commercially available product sizes leaves a significant gap in larger sizes, which are appropriate for effective prevention in older patients and individuals with oral diseases [33].

Standardised and simple uniform labelling may positively impact dental education, patient communication, and oral health behaviour, including compliance. The development and design of a standardised set of interdental brushes that covers all sizes and is suitable for patients of all ages, including healthy and diseased individuals, is recommended.

### 3.2 Clinical Application

#### 3.2.1 Why Should Teeth Be Cleaned Interdentally?

Toothbrushing alone does not effectively reach the interdental spaces between adjacent teeth, leaving portions of the teeth inadequately cleaned. This situation is created primarily by the interdental gingiva filling the space between two teeth apical to their point of contact, creating a ‘sheltered’ area that is challenging to access in normally positioned teeth and even more so in malposition. When initial inflammation of the papillae exposes the interdental area, it creates local conditions conducive to the establishment and growth of bacterial plaque. Therefore, measures for interdental plaque control should be selected to complement plaque control by toothbrushing [35]. The removal of plaque from these surfaces remains a valid objective because in patients susceptible to periodontal diseases, gingivitis and periodontitis are usually more pronounced in these interdental areas than in the oral or facial aspects [36]. Therefore, interdental plaque removal, which cannot be achieved with a toothbrush, is crucial for most patients. In this regard, interdental cleaning is central to the prevention of caries and periodontal disease and its ongoing management and treatment.

#### 3.2.2 When Should Teeth Be Cleaned Interdentally?

There is insufficient evidence to make a definitive statement regarding the optimal timing for the use of interdental aids, whether before or after toothbrushing. A systematic review on the influence of flossing before or after brushing on reductions in the plaque index concluded that there is no significant difference [37]. If indicated, it is recommended to use interdental devices at least once a day, with the choice of ‘when’ largely depending on individual preference, anecdotal evidence and common sense. An individually adapted order can help develop a habitual routine. Moreover, interdental devices should not be combined with toothpastes [38, 39]. Due to the combination of abrasive toothpaste ingredients and the pressure applied during insertion, exposed root surfaces are at particular risk of mineralised tissue material loss [38].

#### 3.2.3 When Is the Ideal Time to Begin Incorporating Interdental Cleaning Into an Oral Hygiene Routine for Optimal Dental Care?

The study by Dos Santos et al. (2011) [40] provides the first point of focus when determining the optimal starting point for interdental cleaning practices. These authors addressed the landscape of recommendations on children's oral hygiene practices and conducted a comprehensive analysis. They aimed to identify and catalogue recommendations from dental and paediatric organisations, assessing how these aligned with current scientific evidence. Topics of interest include toothbrushing frequency, toothbrush design and replacement, flossing practices and considerations regarding the type and amount of toothpaste. In total, 52 of 59 (88%) organisations responded to their request. Regarding the use of floss, the recommended starting age was around 6 months but varied within an advised range of 8–10 or 10–11 years. The suggested frequency of flossing also varied, ranging from daily to two times a week, emphasising that both children and parents play a role in this practice. Several of the interdental hygiene messages identified showed inconsistencies across the various organisations, and although some of these messages align with the best currently available scientific evidence, most lack scientific support.

In examining recommendations provided by dental care associations in greater detail, recent publications were considered, including the American Dental Association (ADA)'s ‘Caring for Little Teeth’ [41]. The ADA suggests initiating interdental cleaning as soon as two teeth are in contact. Specifically, for younger children, they recommend gently cleaning the spaces between adjacent teeth once a day using dental floss, floss holders or tiny brushes [42]. The existing low‐level evidence aligns with the theory that consistent and thorough flossing by a dental care professional may significantly reduce the risk of interproximal caries in young children. However, the most compelling evidence indicates that this benefit only applies in the case of non‐sufficient fluoride exposure [42]. Improved toothbrushing and/or increased topical fluoride exposure could diminish or negate the impact of flossing.

Consequently, the Royal Dutch Dental Association (KNMT) in collaboration with the Dutch Society for Pediatric Dentistry (NVvK) [43] asserts that there is no evidence supporting the caries preventive effect of daily flossing in young people. It is also stated that when an oral care provider recommends using floss, it is vital to instruct patients in its proper use to ensure a high standard of flossing.

Corroborating this, the Dental Health Foundation in Ireland [44] also advises against the general use of interdental cleaning aids for children unless specifically recommended by a dentist. On their website, the Foundation recommends initiating interdental cleaning daily in early adulthood to maintain oral freshness and health while emphasising the potential for damage of the gingival tissues due to incorrect usage until all permanent teeth have emerged.

In summary, the existing literature and recommendations do not provide a consensus on the optimal age for initiating interdental cleaning. The need for interdental cleaning depends on individual factors and oral health. One important consideration is that from a young age children should be taught how to brush their teeth according to the basic oral hygiene advice of brushing twice a day for 2 min with fluoride toothpaste. Professional guidance on oral hygiene should focus on correct brushing systematics and technique. Additionally, oral care providers should advise on an individual basis when to start interdental cleaning in relation to oral hard and soft tissue health. This advice may be given if early signs of tooth decay or periodontal diseases are present, and it can be provided at a young age or later in life. For instance, research suggests that gingivitis tends to become more apparent around puberty, likely due to changes in plaque composition, immune response, gingival morphology and hormonal shifts associated with this period [45]. From an epidemiological perspective, a significant portion of the population should start interdental cleaning in the permanent dentition if early signs of tooth decay or periodontal diseases occur from around the age of 18–20.

#### 3.2.4 How Can Dental Professionals Motivate and Increase Patient Adherence?

##### 3.2.4.1 How Is Oral Health Behaviour Influenced in General?

Health behaviour like personal oral hygiene needs to become a habit over time to unfold its potential benefits. A habit is characterised as something a person does without much conscious planning or thinking. Habitual behaviour is easily triggered by related cues. To form a habit in health behaviour, intrinsic motivation and self‐identity—that is, performing in a manner congruent with how one wants to be—are crucial, as health‐related rewards are often not immediate or hedonistic enough to directly reinforce everyday behaviour [46]. Habit formation is not always linear, but may plateau or even decrease after initial gains, especially when considering that goals of habit formation can differ (e.g., quantity vs. quality of a behaviour) [47]. On a practical level, piggybacking new behaviour into existing routines may be especially helpful to habit formation (i.e., flossing after toothbrushing [48], or substituting a habitual unhealthy behaviour with a healthier one) [47]. Furthermore, the process of habit formation and health behaviour can vary based on individual differences, including factors such as conscientiousness, neuroticism, trait self‐control or preference for routine [49]. Hence, habit creation should ultimately be personalised to the individual.

Several patient variables influence individual oral health behaviour. One important area relates to psychopathology. Common and severe mental illness and dental fear are related to poor oral health [50, 51, 52] and depression [53, 54]. Furthermore, tooth loss and depression may be interrelated [55, 56, 57]. However, other areas of psychopathology have received less attention in dentistry [58, 59]. Psychopathology is not independent of factors such as low socio‐economic status, maladaptive coping strategies, smoking or other substance abuse, or medication effects. Besides psychopathology, other psychologic variables might influence the oral hygiene behaviour, such as intention to practice oral hygiene, self‐efficacy, coping planning, social influence, action planning, attitude, sense of coherence, self‐esteem and locus of control [60, 61]. Distal relational factors such as psychological attachment [62, 63] can also contribute to oral health behaviour, service utilisation, and dental status [64, 65, 66]. While it is safe to say that psychological variables are clearly related to oral health behaviour, more studies with larger samples, longitudinal designs and the inclusion of other variables are needed to gain more confidence in specific factors and associated mechanisms.

##### 3.2.4.2 How Can Dental Professionals Motivate Their Patients to Prioritise Oral Health Care?

Habit formation is important for oral health behaviour, including interdental cleaning. Oral hygiene interventions related to information, instruction, motivation and the training of facilitators may have an impact on dental variables in general [67], especially for individuals with mental illness [68, 69]. However, the existing evidence is heterogeneous regarding methods and results [70, 71]. Low‐quality evidence exists for the benefit of any one‐to‐one oral hygiene advice [72], and there are mixed results for motivational interviewing [73, 74]. In addition, some interventions draw more on general psychotherapy models than prevention [75] or address other risk factors such as smoking [76]. In general, active interventions seem to be helpful, and inter‐individual differences seem to matter to a certain degree [77]. Taken tocher, several intervention programs exist, along with protocols from dentistry and other fields [78], and there are many ways to approach behavioural change [79].

To improve specific techniques such as interdental cleaning it is important to establish a broader strategy to increase personal dental awareness and self‐care. Taking care of one's own mouth should become the norm. While the usual reason for prevention is related to disease, it may be helpful to reverse this approach with the overall goal of shaping a more positive relationship with the oral cavity. This positive change includes known cultural factors such as the role of white teeth in body image [80] and accepting inter‐individual differences in gum bleeding and oral odour as a starting point to form curiosity and a positive motivation and intention for interdental care. If the focus of oral health behaviour shifted in this manner, it would also allow clinicians to be more flexible with changing evidence regarding recommendations for starting interdental care while encouraging patients to adopt the habit of reserving time for related behaviour. Such a change would also allow the setting of realistic goals for maintaining healthy teeth, would bring immediate hedonistic rewards, such as fresh breath after brushing, and would increase intrinsic motivation, which are important for habit formation. Hence, interventions should become more playful and include a variety of approaches such as apps, storytelling, peer support and facilitators.

Oral hygiene needs to become more thorough in cases of dental diseases, and patients need more professional support. Motivational factors also change as patients become aware of their oral health problem. In cases of mental diseases, cooperation is required between dental professionals and mental health specialists.

##### 3.2.4.3 How Do Individual Choose an Approach for Behaviour Change? A Practical Primer

Considering the various approaches to behaviour change mentioned above [81], and with studies showing that different approaches or devices often affect subjective feelings more than tooth‐related outcomes, guided choice may enhance adherence through improved self‐efficacy or self‐control. In addition to known prevention approaches such as oral health‐related goal‐setting, planning, and self‐monitoring, also known as GPS [82], it may be advisable to consider distal psychological variables that relate to the individual and the dentist–patient alliance in addition to the actual cleaning. This approach can help patients develop self‐awareness and insight into their needs and related behaviour to facilitate long‐term change. As several interventions seem to work, approaches from different theoretical backgrounds can be applied in accordance with the dentist's or patient's preference and practical considerations. Patient involvement in the development of new interventions or the refinement of existing ones is essential.

### 3.3 Specific Patient Groups

#### 3.3.1 Interdental Cleaning for Patients With Healthy Teeth and Periodontal Tissues

The supracrestal tissue attachment, historically termed ‘biologic width’, is a histological concept introduced by Cohen [83] and further defined by Ingber et al. in 1977 [84]. The concept encompasses the apico‐coronal height of the junctional epithelium and the supracrestal connective tissue attachment [85]. Several studies have shown that the most consistent aspect is the vertical dimension of the connective tissue attachment, while the junctional epithelium exhibits more variability [86]. Under healthy circumstances, the junctional epithelium extends to the contact point, completely occupying the approximal spaces. Essentially, oral microorganisms are excluded from the approximal space and biofilm formation is not possible. Interdental cleaning devices, if employed, can disrupt the junctional epithelium, necessitating daily interdental cleaning. However, caution is advised when recommending the initiation of interdental cleaning in healthy sites without apparent attachment loss and/or demineralization, as it may lead to trauma. Although the risk of trauma may be lower with the use of oral irrigators [87, 88], their cost–benefit ratio should be carefully considered. Professional guidance is essential to ensure optimal effectiveness and to prevent potential harm [3]. In balancing the risks and benefits, interdental cleaning is not advised as long as the interdental space is properly filled and safeguarded by a healthy interdental papilla. This approach also supports the strategy for targeting the ideal time to begin incorporating interdental cleaning into oral hygiene routines for optimal dental care (3.2.3). Hence, it is essential to check individuals regularly to detect the initial signs of disease and initiate interdental self‐care.

#### 3.3.2 Interdental Cleaning in Patients With Gingivitis

Effective plaque removal is fundamental for preventing gingivitis and for the primary and secondary prevention of periodontitis. Preventing gingivitis, viewed as part of the same continuum of inflammatory disease as periodontitis, may significantly impact periodontal care [89, 90, 91]. Prevention involves educational interventions, self‐performed plaque removal and professional removal of plaque and calculus. Successful oral hygiene requires motivating the patient, providing adequate tools and offering professional instruction.

Diligent oral hygiene practices reduce dental plaque along the gingival margin and alleviate stress on associated tissues. Oral hygiene is a non‐specific means of reducing plaque mass, benefiting inflamed tissues adjacent to bacterial deposits [92]. Maintaining an optimal supragingival plaque control regimen can influence the composition of the periodontal pocket microbiota and modify the quantity and composition of subgingival plaque [93].

In populations that use toothbrushes, the interproximal surfaces of the molars and premolars are the predominant sites of residual plaque [31]. The selection of an appropriate interdental cleaning aid is influenced by several factors, including ease of use, size of interdental space, acceptability, dexterity and motivation of the individual [18]. While there is no one‐size‐fits‐all solution, a desirable interdental cleaning tool should be user‐friendly and efficient in plaque removal and should not harm soft or hard tissues. Environmental friendliness is an aspect that has received more emphasis in recent years [94, 95].

Sälzer et al.'s (2015) [13] meta‐review on the efficacy of interdental mechanical plaque control in managing gingivitis provided the underlying evidence for the recommendations of the European Federation for Periodontology [3]. The authors' synthesis of systematic reviews concluded that interdental cleaning with interdental brushes is the most effective method. In contrast, most of the included studies did not provide substantial evidence supporting the general effectiveness of flossing in plaque removal. Therefore, floss use is not recommended, except for sites of gingival and periodontal health where interdental brushes may not pass through the interproximal area without causing soft tissue trauma [13].

Recent studies show that OI offer additional plaque removal and improved gingival health compared to brushing alone, either with a manual or powered toothbrush. In cases of gingivitis, combining brushing with oral irrigation is more effective at reducing gingival bleeding than brushing with flossing [24, 96, 97].

The introduction of non‐wired interdental cleaning devices (NWICD), such as rubber/elastomeric interdental cleaning sticks, two decades ago has produced a notable surge in the market in terms of sales and variety. The evidence suggests that this device should be recommended [19]. Furthermore, patient preference and acceptance of this device are high, and it can serve as a starting point for novice interdental cleaners. Despite its intuitive use, personalised instruction and follow‐up by a dental care professional are essential for optimising the benefits of NWICD. While some evidence supports its efficacy for gingivitis patients [19] further research is needed to establish its effectiveness in the maintenance phase for other frequent user groups, such as periodontitis patients and those with dental implants.

Contrary to common misconception, the presence of bleeding does not necessarily warrant avoiding interdental cleaning; rather, it signals inflammation requiring appropriate treatment [98]. Self‐feedback on gingival bleeding is the fundamental concept of the Eastman Interdental Bleeding Index [99]. Gingival bleeding during interdental cleaning may also indicate trauma, such as lacerations or gingival erosions, or serve as an indicator of inflammation.

#### 3.3.3 Interdental Cleaning and Patients at Risk of Caries

Several studies have investigated the relationship between interdental cleaning and caries. A systematic review from almost two decades ago concluded that professional flossing in children with low fluoride exposures is the only highly effective method for reducing interproximal caries risk. Therefore, caution should be exercised when extrapolating these findings to more typical floss users, as self‐flossing has not demonstrated a significant effect [42]. Marchesan et al. (2018) [100] found that interdental cleaning is associated with lower levels of caries and fewer missing teeth. In a sample comprising almost 6000 patients, Pitchika et al. (2021) [101] reported that, over the long term, the use of power toothbrushes and interdental cleaning aids contributed to an increased number of caries‐free healthy surfaces and teeth in adults and seniors. The large effect of fluoride on caries prevention and the regular use of fluoride dentifrice may be another reason why interdental devices' efficacy is difficult to prove [2]. Thus, the collective findings underscore the nuanced role of interdental care practices regarding caries across diverse populations, and advice should be tailored according to the individual's oral health status and caries risk profile.

#### 3.3.4 Interdental Cleaning and Patients With Periodontitis

A randomised clinical trial was recently performed to determine the efficacy of several protocols involving adjunctive interdental devices in periodontitis‐affected patients with interdental recessions [102]. The authors concluded that dental floss did not add any benefits in terms of plaque and gingival inflammation when compared to toothbrushing alone. This situation might be due to patients' manual dexterity and lack of compliance with regard to flossing [103]. Moreover, the interdental attachment loss in patients with periodontitis may expose part of the root structure, which often presents with concavities and grooves, which can diminish the efficiency of flossing. Placing a knot in a string of floss may improve the efficacy [104].

Almost half a century ago, based on ex vivo data, interdental brushes emerged as the optimal tool for cleaning between teeth, especially for individuals with periodontal diseases. Consistent use of interdental brushes enables individuals to maintain supragingival proximal surfaces free of plaque and effectively removes subgingival plaque below the gingival margin, reaching depths of 2–2½ mm beneath the gingival margin [105].

Slot et al. (2008) [14] conducted a systematic literature review to assess the efficacy of interdental brushes as adjuncts to toothbrushes in patients with gingivitis or periodontitis, focusing on plaque and clinical parameters of periodontal inflammation. Most of the reviewed studies demonstrated a significantly positive difference in plaque index when using interdental brushes compared to floss. This finding is reinforced by additional sources [13], including the most recent Cochrane review by Worthington et al. (2019) [16] and two Bayesian network meta‐analyses [26, 106]. Collectively, this evidence provides robust support for the effectiveness of interdental brushes as the preferred method for interdental plaque removal. This conclusion was confirmed in a network meta‐analysis with multiple parameters of gingival inflammation, which used two correlated outcome variables, the Gingival Index (GI) and bleeding on probing (BOP) [27].

A recent systematic review [17] focusing on periodontal maintenance patients revealed a limitation in available studies that met the inclusion criteria for various oral hygiene devices. The resulting evidence was characterised by low certainty, preventing the formulation of strong evidence‐based conclusions regarding the effectiveness of any specific oral hygiene device for patient self‐care in periodontal maintenance. Notably, only for IDBs used as an adjunct to toothbrushing did a small yet significant effect on the Plaque Index (PI) emerge, albeit derived from indirect evidence. The overall findings underscore the need for further research and a cautious interpretation of the available evidence when assessing the efficacy of interdental hygiene devices in the context of periodontal maintenance [17].

#### 3.3.5 Interdental Cleaning and Patients With Implants and/or Peri‐Implantitis

Maintenance of optimal oral hygiene by patients and adequate supportive implant maintenance by dental professionals are key to preventing peri‐implant disease occurrence [107]. Patient‐administered mechanical plaque control is recognised as the standard care required in peri‐implant disease management [108]. However, notable gaps exist in the evidence for the most effective self‐performed oral hygiene practices regarding dental implants. Current home care recommendations rely on available knowledge related to natural tooth‐care [109]. Distinct anatomic features of marginal gingival tissues compared to natural teeth introduce complexities. Furthermore, implant position, diameter, mucosal emergence depth and diverse implant‐supported prosthetic designs are considered important factors for oral hygiene considerations for dental implants. In particular, size discrepancies between the restoration and the implant may promote the creation of niches, leading to restricted accessibility for sufficient interproximal oral hygiene and predisposition of peri‐implant diseases. Studies have shown that implant restorations with a wide emergence angle or convex profile are considered a risk indicator for peri‐implant diseases, especially for bone‐level implants [110, 111, 112]. Furthermore, exposed rough surfaces of dental implants may compromise peri‐implant conditions, making dental floss use potentially problematic [113]. Hence, the authors of this paper strongly advocate for well‐executed clinical trials to explore various aspects of oral hygiene around dental implants.

#### 3.3.6 Interdental Cleaning and Patients With Fixed Prosthetic Tooth Replacement

The distribution of soft deposits (i.e., plaque) on abutment teeth is similar to that on uncrowned homologous teeth [114]. The pattern of this distribution on the different surfaces of the abutment teeth does not seem to deviate from the general distribution of plaque on teeth, meaning that the interproximal surfaces accumulate more and are the most frequent sites of soft deposits [115]. Furthermore, the approximal areas show the most severe lesions and are the most frequent sites of periodontal disease.

Restorations commonly extend into the pocket, and such subgingival restorations are known to accumulate plaque. From their cervical margins, the plaque is likely to proliferate apically, which may cause periodontal disease [105]. The interdental brush can remove plaque both supra‐ and sub‐gingivally. It is suggested that, if possible, final restorations should be shaped to allow access to the interdental brush. Moreover, patients should be instructed on how to use the brushes [105].

There is a noticeable scarcity of information in the existing literature regarding the use of various interdental cleaning devices in patients with FPD. At present, the presumption is that the recommendations for interdental cleaning of teeth with FPD are the same as those for natural teeth.

#### 3.3.7 Interdental Cleaning and Patients With Fixed Orthodontic Appliances

Fixed orthodontic brackets contribute to plaque buildup and bacterial colonisation, increasing gingival inflammation and bleeding [116]. Individuals undergoing fixed orthodontic therapy frequently exhibit suboptimal plaque control, primarily due to challenges in performing effective oral hygiene practices with orthodontic appliances in place. A Cochrane systematic review compared the effectiveness of using standard toothbrushes alone to a combination of standard toothbrushes and interdental brushes for plaque removal during fixed orthodontic appliance therapy [117]. No eligible studies related to the objectives of their review were identified.

The wear of interdental brushes and normal toothbrushes is increased while cleaning orthodontic appliances; hence, the brushes need to be replaced frequently. This necessity increases the economic burden of oral hygiene products for this group of patients [117]. Based primarily on this cost–benefit analysis and not so much on efficacy, the review authors concluded that ‘the present practice of recommending the use of interdental brushes in addition to standard toothbrushes is not supported by clinical investigations*’*. Furthermore, the authors commented that well‐designed RCTs are required to provide evidence for determining best clinical practice in this area. In addition, a 2008 study demonstrates that an oral irrigator equipped with a specialised orthodontic jet tip leads to improvements in plaque and bleeding scores in adolescents with fixed orthodontic appliances [118]. In contrast, a more recent study found no evidence to support the benefits of using an oral irrigator in addition to a manual toothbrush for patients wearing fixed orthodontic appliances [25].

#### 3.3.8 Interdental Cleaning in Special Needs Patients

‘Special needs dentistry’ or ‘special care dentistry’ involves the dedicated effort to enhance oral health for individuals and groups who experience intellectual, mental, physical, sensory, medical, emotional, or social impairment or disability, often encompassing a combination of several of these factors [119]. Patients with special healthcare needs exhibit a higher prevalence of oral disease, including caries and periodontal diseases [120]. Furthermore, the increased risk of susceptibility to caries and periodontal disease may be influenced by genetic or medical conditions and the use of prescription medications or recreational substances [119].

A systematic review concerning the oral health of adults with learning difficulties found that individuals in this particular group experienced a higher incidence of untreated carious lesions, poorer oral hygiene practices, and an increased prevalence and severity of periodontal disease than the general population [121]. In addition, in a study examining the oral health status of almost 5000 adults with learning and developmental disabilities reported an overall periodontitis prevalence of 80.3%. Although many of the younger individuals in the study did not experience periodontitis, a significant proportion exhibited gingivitis [122].

Research indicates that individuals with Down's syndrome exhibit a higher prevalence of missing teeth, increased bleeding on probing, and elevated gingival and plaque indices [123, 124]. Other researchers have demonstrated that these individuals are more prone to developing periodontal disease [125]. A meta‐analysis examining the oral health status of children with autism concluded that children with this condition have an elevated risk of caries prevalence and poor oral hygiene and exhibit a lower salivary pH [126].

Bensi et al.'s (2020) meta‐analysis on oral health in children with cerebral palsy revealed that they appear to have an increased risk of caries in the primary dentition, poorer gingival status, Angle Class II malocclusion and anterior open bite [127]. Physical impairments such as Parkinson's disease, multiple sclerosis and rheumatoid arthritis are associated with more severe periodontal disease. Patients with Parkinson's disease exhibit poorer oral health, including greater gingival recession, deeper periodontal pockets, increased bleeding on probing, tooth mobility and inferior oral hygiene [128]. Concurrently, rheumatoid arthritis is associated with an increased occurrence of periodontal disease [129]. Impairments like finger stiffness, joint swelling, finger deviation and pain can affect the ability to maintain regular self‐care practices, including toothbrushing and interdental cleaning.

The aging population presents a growing healthcare challenge with the onset of cognitive impairment in older adults. US and Finnish surveys have independently linked reduced cognitive function to increased odds of periodontal disease and a higher incidence of carious teeth, edentulousness and poor denture hygiene [130]. A recent systematic review focusing on oral health status and the need for oral care in an aging population concluded that older individuals with dementia exhibit higher levels of plaque, coronal and root caries, retained roots, gingival issues and periodontal disease. The poor oral health observed in this group can be attributed to reduced submandibular salivary flow, cognitive decline, and deterioration in motor and communication skills [131]. However, an increasing proportion of less impaired seniors, as well as older people retaining natural teeth, is anticipated in the coming years. For this population, periodontal disease and fixed or removable dentures and implants are often present, making consistent interdental hygiene critical.

Groups facing social disadvantages, including the homeless, migrants, prisoners, individuals with low socio‐economic status and those with mental illness, exhibit inferior oral and overall health compared to the general population [132]. Consequently, healthcare requirements are heightened within these groups, although they simultaneously face greater challenges in accessing care.

The patient's or caregiver's motivation, their ability to understand and maintain proper oral hygiene and any physical or medical limitations that may hinder home care should be considered, potentially necessitating education and training for the patient and caregiver [133]. Barriers to maintaining adequate oral hygiene include disinterest, a cariogenic diet preference, loss of tactile ability, physical disabilities, learning difficulties and a predominant focus on the patient's disability at the expense of oral health needs. External factors such as limited time, frequent caregiver changes, prioritising more urgent needs [134], limitations of the national healthcare system, shortcomings of the insurance system and insufficient funding for oral hygiene support in care homes also contribute to these challenges [135].

The importance of oral hygiene in special needs patients is paramount, especially for individuals and groups facing various health problems. Given the high prevalence of oral diseases in these patients, strict oral hygiene protocols, including interdental cleaning, are essential. However, there is currently little evidence on the efficacy and compliance of interdental cleaning in patients with special needs, which underlines the need for future research.

## 4 Recommendations Emerging From the Round Table

General guidelines for interdental cleaning are provided below, addressing aspects such as the rationale, target population, effective techniques and tools (Table 1). Subsequently, specific interdental hygiene recommendations are provided focusing on various factors, encompassing conditions, life stages, implants and dental prosthetics and devices. Table 1 gives a visual summary of these recommendations, consolidating the key points from the roundtable discussions.

### 4.1 General Recommendations for Interdental Cleaning

#### 4.1.1 Basic Oral Hygiene Advice

- Toothbrushing twice daily with fluoride toothpaste, for at least 2 min, using a toothbrush [136].
- Expelling excess toothpaste and rinsing with a small amount of water [137, 138].

#### 4.1.2 Rationale and Target Population for Interdental Cleaning: Who and Why

- As long as the interdental space is filled and protected by a healthy interdental papilla, there is no need for interdental cleaning. However, when interdental tooth surfaces are exposed to the oral cavity they will be covered by biofilm, which requires daily plaque removal. Based on epidemiologic data, this combination is likely to be present in most adults above the age of 18 [46]. Depending on early clinical signs and symptoms of caries and periodontal disease, younger individuals may also need selective interdental cleaning on these surfaces.

#### 4.1.3 Effective Techniques and Tools for Interdental Cleaning: How

- Once the papilla no longer fills the interdental space, toothbrushing alone cannot adequately reach into the interdental spaces to clean these surfaces effectively in case of diseased papillae or in open interdental spaces. Therefore, adjunctive oral hygiene devices are necessary, and these should be integrated into an individual's daily routine.
- A wide array of interdental cleaning devices is currently available. The prevailing evidence supports interdental brushes as the most effective option. However, it is necessary to consider individual abilities, motivation and intra‐oral circumstances when adopting this recommendation to achieve an optimal level of plaque removal. Additionally, to enhance adherence, it is important to consider individuals' preferences.
- Recent decades have seen significant advancement and diversification in interdental cleaning devices. Moreover, there is a noticeable increase in public awareness regarding interdental cleaning, as indicated by market sales trends. The local availability and financial resources should also be taken into account. Dental care professionals should be well‐informed about the current range of options available to provide tailored recommendations for their patients.

#### 4.1.4 Interdental Cleaning Devices and Toothpaste

- Interdental cleaning devices should not be used in combination with toothpaste, and this applies particularly to IDBs and potentially to NWICD. These can both be used with non‐abrasive therapeutic gels [139].

#### 4.1.5 Considerations for the Sequence of Interdental Cleaning and Toothbrushing

- The order of use: interdental cleaning device <> Toothbrushing lacks consistent evidence regarding the effectiveness of oral hygiene procedures in a specific sequence. Patient preference becomes pivotal in the absence of robust evidence, particularly concerning needs‐related oral hygiene. It is important to note that interdental cleaning and toothbrushing can be considered separate procedures. Maintaining a consistent order is advisable for habit formation.

#### 4.1.6 Replacement of Interdental Brushes

- Clear guidelines for replacing interdental brushes are lacking, and the decision is contingent on factors such as deformation, including bending of the metal core, bending of the filaments (which is also influenced by filament diameter) and loss of filaments.

#### 4.1.7 Optimal Number of Recommended Interdental Brushes

- Expert opinion suggests initially not using more than two to three different sizes of interdental brushes; if additional sizes are required, consider recommending conically shaped brushes or brushes with thinner filaments, covering a wider range of interdental space sizes.

#### 4.1.8 Interdental Device Instructions

- Experts agree that the oral hygiene instructions provided are often not very clear or illustrative. Those in the Lindhe textbook 7th edition [140] are the most appropriately described. The instructions are helpful for training and alignment among dental care professionals, enabling them to provide information and instructions for patients. For the updated and adjusted version of this text, see online Appendices S2–S6.

### 4.2 Specific Oral Hygiene Recommendations

#### 4.2.1 Gingivitis

In addition to the basic oral hygiene advice, if the interdental space allows, interdental brushes are the first choice.

#### 4.2.2 Periodontitis, Reduced Periodontium and Proximal Root Caries

Along with basic oral hygiene advice, interdental brushes are the preferred option. It is necessary to consider and respect patients' preferences in selecting the most suitable interdental cleaning method. Additionally, alongside interdental care, implementing caries preventive measures is essential in maintaining optimal oral health for individuals in these groups.

#### 4.2.3 Dental Implants

It is essential to ensure dental implants are provided with restorations that allow effective interdental cleaning. Currently, there is limited evidence‐based recommendations specific to implant care. As a result, present home care suggestions founded on existing knowledge are related to the cleaning of natural teeth [141].

#### 4.2.4 Peri‐Implant Mucositis

In addition to basic oral hygiene advice, interdental brushes are typically the primary recommendation for effective plaque removal. However, in instances where the interdental space or crown contour does not permit the use of interdental brushes, alternative devices like (super) floss or oral irrigators should be taken into consideration.

#### 4.2.5 Peri‐Implantitis

When addressing peri‐implantitis, the interdental cleaning recommendations remain consistent with those for peri‐implant mucositis. However, in the case of peri‐implant bone loss, floss use should be avoided as it may attach to the rough surface of the implant and potentially exacerbate the condition [113].

#### 4.2.6 Removable Prosthesis

For cases involving double crowns, it is advised to ensure that interdental cleaning can be facilitated with the secondary part in position as per the design. Interdental brushes should be prioritised as the primary cleaning tool in such scenarios.

#### 4.2.7 Orthodontic Patients

Basic oral hygiene advice is applied, encompassing the recommendation to incorporate interdental cleaning based on clinical indicators of caries, gingivitis and periodontitis. Additionally, interdental brushes play an indispensable role in cleaning intra‐oral orthodontic appliances, particularly when manoeuvred between the wire and tooth surface. Cleaning of a removable appliance (such as clear aligners) should not be forgotten.

#### 4.2.8 Children

Parents serve as role models in promoting optimal oral health habits in children. Demonstrating interdental cleaning practices based on clinical signs and symptoms of caries, gingivitis and periodontitis allows children to understand its significance within their personal oral care routines. Specific approaches may be considered based on individual needs. For instance, in cases where interdental cleaning is warranted during deciduous dentition, the use of floss applied by parents is recommended as a first‐line choice. For those who are starting with interdental cleaning, NWIDC provides a gentle introduction to this aspect of oral hygiene. In situations involving hypomineralisation, interdental brushes are suitable if tooth sensitivity allows this.

#### 4.2.9 Adolescents and Adults

In cases of clinical signs and symptoms of caries, gingivitis and periodontitis, in addition to the basic oral hygiene advice, interdental brushes take precedence as the primary tool of choice. For novice interdental cleaners, NWIDC serves as a gentle introduction. Furthermore, in cases where interdental brushes may cause discomfort or soft tissue trauma, the recommendation of flossing is relevant.

#### 4.2.10 Older and Special Needs Patients

In addition to the advice for adolescents and adults, special consideration must be given to older and special needs patients. Educating caregivers should encompass comprehensive training in oral hygiene practices (i.e., effective oral hygiene techniques and instruction). Whether the patient is capable of performing oral self‐care or relies on a caregiver, factors like dexterity need to be taken into account. In situations where dexterity is limited, prioritising ease of use over effectiveness becomes crucial. For instance, in patients with no swallowing problems and the ability to spit, oral irrigators may be recommended for their user‐friendly approach.

## 5 Discussion

There are potential barriers to implementing the above‐mentioned recommendations. The challenges and corresponding solutions are addressed below, spanning the population level and the dental care profession.

### 5.1 Population

#### 5.1.1 Information and Awareness

To enhance information and awareness, the development of educational content that is easily translatable and endorsed by professional societies should be developed. This content will serve as a means to disseminate important information on interdental cleaning across diverse populations through professional societies, public platforms, various social media channels and advertisements. This communication also includes channels such as glossies, semi‐professional publications, educational AI systems focused on interdental cleaning, updates on Wikipedia and platforms dedicated to interdental cleaning challenges. In essence, this implies advocating for the utilisation of all available channels to maximise the message's reach and impact.

Although the evidence is clear, advice for interdental hygiene varies between different dental associations. This divergence may be due to the fact that socio‐economic conditions, availability of devices and cultural traditions are relevant confounders in the acceptance of different approaches [30]. There is also a high dynamic market with new developed devices in that field. Some, like non‐wire interdental cleaning tools, are widely accepted by consumers but lack adequate scientific clinical evidence and, therefore, are not included in recommendations to date. This situation is confusing for the consumer. Therefore, this paper contributes to providing more practical, evidence‐based instructions that are accessible to populations in industrialised countries

#### 5.1.2 Requirement for a Behavioural Shift, Which Is Generally Challenging to Attain

Integrating interdental cleaning into a broader framework of dental self‐awareness and self‐care is essential, with habit formation being important to lasting behavioural change. Strategies must be tailored to the severity of the disease and the specific conditions present. These conditions can encompass factors at the level of an individual tooth or implant, within the oral cavity (e.g., orthodontics), or among specific patient groups (e.g., children, geriatrics). Screening for physical and/or mental conditions, preferences and adherence rates, along with effective communication with other medical and health professionals, is crucial. Recognising the diverse variables at play and providing patients with a range of choices can empower them to take an active role in their oral health.

#### 5.1.3 Individual Optimum

Achieving the individual optimum in interdental cleaning, tailored to the unique needs and capabilities of each patient, is a formidable challenge in daily dental practice. Attaining this requires careful consideration of oral health factors and the patient's personality. The aim is to attain an individualised standard of care, recognising that this benchmark may evolve over time and differ from the ideal. Striking the right balance between motivation and frustration due to excessive demands is imperative. In addition to efficacy, personal preferences and manual dexterity can lead to recommending alternative interdental cleaning devices with the aim of reaching the individual's optimum. Studies have demonstrated the efficacy of oral irrigators [142] and NWIDC [19] as reasonable alternatives to interdental brushes in patients without periodontitis. Within the framework of a long‐term care plan, the dental care professional's role transcends mere instruction, transforming into a coaching and guiding partnership that enables the patient to independently and effectively manage their oral self‐care regimen to attain the individual optimum.

#### 5.1.4 Insufficient Dexterity to Effectively Utilise the Devices

To address the issue of insufficient dexterity, it is necessary to focus on enhancing the usability of available oral hygiene products. This process may involve personalised handles for oral hygiene devices, allowing a more tailored and manageable approach to maintaining oral health. In some cases, the importance of ‘ease of use’ over sheer efficacy may be warranted, especially for individuals facing challenges with dexterity. For example, opting for an oral irrigator over an interdental brush can improve the individual's optimum oral self‐care. Furthermore, daily oral hygiene routines should be adapted to what is most feasible for the individual.

#### 5.1.5 Expense Considerations in Daily Oral Care

To economise costs, it is recommended not to choose interdental single‐use cleaning devices. This approach helps reduce expenses and promotes sustainability by minimising waste. Additionally, depending on the required level of care, patients may be presented with specialised options, including considerations related to insurance coverage.

#### 5.1.6 Pain, Gingival Bleeding and Discomfort When Using Interdental Cleaning Devices

For those beginning to use interdental cleaning devices, it is normal to initially experience increased bleeding. Furthermore, improper use can lead to soft tissue trauma and result in discomfort. It is important to provide patients with thorough and precise instructions and information beforehand. Following a recommendation for an interdental cleaning device, dental care professionals should conduct follow‐up assessments with patients to evaluate the correct usage and the clinical effectiveness of the devices.

### 5.2 Dental Care Professionals

#### 5.2.1 The Lack of Intra‐ and Interprofessional Alignment in Procedures and Recommendations in the Promotion of Interdental Care

To address this issue, professional and periodontal societies should be contacted to work toward aligning the recommendations they provide for the population and individual patients, based on the available evidence. Furthermore, interdental cleaning should be a focal point in dental care practices and at the level of the individual dental care professional.

The existing guidelines lack specificity and practicality, emphasising the need for clear recommendations on the use of interdental cleaning devices. This guidance should be tailored to various patient‐related factors. There is also a deficiency in education and awareness regarding the efficacy of interdental cleaning devices, which calls for the incorporation of evidence‐based recommendations into dental curricula.

#### 5.2.2 Dental Care Professionals' Lack of Time, Knowledge, and Motivation to (Re)instruct Patients in Interdental Cleaning

The high efficacy of interdental cleaning, particularly with interdental brushes, necessitates prioritising it in chairside time management. Prevention should be an integral component of the long‐term ‘plan of care,’ with the dental care professional and the patient sharing responsibility. Given the constraints of limited time, emphasis should be placed on the most aspects of interdental cleaning. Additionally, the provision of supportive, correct and modern educational content, facilitated by industry and professional societies, can contribute to reducing chair time. For instance, detailed instructions like the ones in Appendices S2–S6 could be provided to clinicians to support their instructions.

## 6 Clinical Relevance

### 6.1 Scientific Rationale for the Study

Given the widespread prevalence of periodontal diseases and dental caries, this summary of a roundtable meeting on interdental cleaning seeks to consolidate evidence for optimising interdental cleaning practices and promoting comprehensive preventive care strategies.

### 6.2 Principal Findings

Our expert meeting provided detailed recommendations for personalised interdental care, highlighting the efficacy of several interdental cleaning devices in preventing caries and periodontal diseases.

### 6.3 Practical Implications

The formulated recommendations offer actionable insights for clinicians, advocating for the integration of interdental cleaning devices into daily routines. By prioritising individualised care based on clinical indicators, this paper facilitates enhanced oral health outcomes across various patient demographics and oral health conditions.

## Author Contributions

All authors approved the final version of this manuscript before submission and agreed to be accountable for all aspects of the work, ensuring that questions related to the accuracy or integrity of any part of the work were appropriately addressed and resolved. **Tim M. J. A. Thomassen:** contributed to the design, analysis and interpretation, and drafted the primary manuscript. **Christian Graetz, Christof E. Dörfer, Dagmar E. Slot, and Fridus (G. A.) Van der Weijden:** contributed to the conception, planning and organisation of the round table discussion and provided critical feedback during the drafting and revising of the manuscript. **Inmaculada Tomás, Nicola Discepoli, Johannes C. Ehrenthal and Cornelia Frese:** provided expert insights and perspectives during round table discussions and reviewed and edited the manuscript for accuracy and coherence. **Sonja Sälzer:** contributed to the design, analysis and interpretation and drafted the manuscript.

## Funding

Funds for this round table were provided in part through an unrestricted educational grant that covered the costs of travel, hotel and consumptions from DENTAID Oral Health Experts. The company had no say in the design of this paper nor did they influence the content reporting and publishing of the findings. This manuscript received no other specific grant from any funding agency in the public, commercial or not‐for‐profit sectors. No other funding was accepted for this study, as work for this paper was funded by regular academic appointments. All participants filed detailed disclosure of potential conflict of interest relevant to the topic. Declared potential dual commitments included having received research funding, consultant fees and speakers' fees from: Colgate‐Palmolive, CP GABA, Procter & Gamble, Johnson & Johnson, Sunstar, Unilever, Philips, Dentaid, Ivoclar‐Vivadent, Heraeus‐Kulzer and Straumann.

## Conflicts of Interest

The authors declare no conflicts of interest.

## Supporting information


**Appendix S1:** Pre‐formulated questions serving as the initial step of the round table discussion.
**Appendix S2:** Interdental brush implementation: recommendations for optimal usage and oral health benefits.
**Appendix S3:** Dental floss implementation: recommendations for optimal usage and oral health benefits.
**Appendix S4:** Non wire Interdental Cleaning Devices implementation: recommendations for optimal usage and oral health benefits.
**Appendix S5:** Woodsticks implementation: recommendations for optimal usage and oral health benefits.
**Appendix S6:** Oral Irrigator: recommendations for optimal usage and oral health benefits.

## Data Availability

Data derived from publicly accessible resources and expert consensus. The data supporting the findings of this paper are based on expert consensus from a round table meeting and from public domain databases, specifically PubMed/Medline and Cochrane‐CENTRAL. These data were derived from analyses and resources available in the original, publicly available publications.
