# Supplementary material for: The Patient‐Centred Interdental Cleaning Concept—Consensus Based on a Round Table
Source: Int J Dent Hyg. 2026 Jan 26;24(2):247–64. doi: 10.1111/idh.70009 (PMC13050390; doi:10.1111/idh.70009)
Supplement: Supplementary file 1 — Appendix S1: Pre‐formulated questions serving as the initial step of the round table discussion. Appendix S2: Interdental brush implementation: recommendations for optimal usage and oral health benefits. Appendix S3: Dental floss implementation: recommendations for optimal usage and oral health benefits. Appendix S4: Non wire Interdental Cleaning Devices implementation: recommendations for optimal usage and oral health benefits. Appendix S5: Woodsticks implementation: recommendations for optimal usage and oral health benefits. Appendix S6: Oral Irrigator: recommendations for optimal usage and oral health benefits. [file IDH-24-247-s001.docx]

**“The Patient-Centered Interdental Cleaning Concept”**

*- Consensus based on a round table –*

Online Appendices

**Online supporting information index**

**Appendix S1**

Pre-formulated questions serving as the initial step of the round table discussion.

**Appendix S2**

Interdental brush implementation: recommendations for optimal usage and oral health benefits

**Appendix S3**

Dental floss implementation: recommendations for optimal usage and oral health benefits

**Appendix S4**

Non-Wire Interdental Cleaning Devices (NWICD) implementation: recommendations for optimal usage and oral health benefits

**Appendix S5**

Woodsticks implementation: recommendations for optimal usage and oral health benefits

**Appendix S6**

Oral Irrigator: recommendations for optimal usage and oral health benefits

**Appendix S1. Pre-formulated questions serving as the initial step of the round table discussion.**

| **Impulse lecture** | **Expert** | **Titel** |
| --- | --- | --- |
| 1 | *Dagmar Else Slot* | Why and when should we clean interdentally? |
| 2 | *Christof Dörfer / Christian Graetz* | What is the recommended methodology to investigate the efficacy of interdental cleaning devices? |
| 3 | *Cornelia Frese* | How to facilitate the standardization of the choice of the interdental brush in the dental practice? |
| 4 | *Fridus van der Weijden* | What to recommend for interdental cleaning for patients with healthy teeth and gums, and for patients with gingivitis? |
| 5 | *Nicola Discepoli* | What to recommend for interdental cleaning for patients with periodontitis, and with implants or peri-implantitis? |
| 6 | *Inmaculada Tomás Carmona* | What are the needs of interdental cleaning in special needs patients – what to recommend? |
| 7 | *Johannes Ehrenthal* | How can dental professionals motivate and increase patient adherence? |

**Appendix S2.** Interdental brush implementation: recommendations for optimal usage and oral health benefits

**Interdental brush implementation: recommendations for optimal usage and oral health benefits**

**How to choose an interdental brush?**

Interdental brushes can be purchased in a variety of sizes, ranging from small to very large. The size of the space between the teeth determines the size of the diameter of the bristles on the brush. Dental care professionals can precisely identify which sizes you need and also demonstrate their proper use. A brush that is too small will not completely clean the interdental spaces, and a brush that is too large can injure the gums. For posterior areas some manufacturers provide grip extensions.

The wire of an interdental brush must be thin and the bristles fine and long. With such dimensions, the interdental brush with the right size will fill the entire space between the teeth quite softly and gently. The size of the interdental brush should be adapted so that the interdental space should be filled completely allowing for adequate insertion force; reaching the marginal areas near the gingiva without a traumatization of the tissue. Some resistance should be felt so that the brush filaments experts sufficient shear force for optimal cleaning ability resistance when the brush is moved back and forth between the teeth.

Tooth spacing varies, so it is often necessary to use different brush sizes within one oral cavity for optimal cleansing. However, for initial recommendations, compromises have to be found to limit the number of interdental brush sizes to 2-3.

Do not be alarmed if the gums bleed initially. This bleeding does not mean that you have an injury but inflammation, which is caused by concealed, old plaque. This reaction is fairly normal during the first week. Using the interdental brush will soon cure this inflammation, and the bleeding will stop. As the inflammation subsides, the interdental spaces will become slightly larger, and you will most likely need a larger interdental brush.

**Instructions*:**

- Always use the interdental brush without toothpaste. Non-abrasive preventive gels like fluoride or Chlorhexidine may be used when needed.
- Hold the interdental brush at the grip or just behind the bristles between the thumb and forefinger. Support can be achieved, when necessary by placing your other fingers on your chin. From the outer side of the space, push the interdental brush carefully between the teeth, taking care that the brush remains at a right angle to the teeth.
- Avoid scraping the center (metal spiral part) of the brush against the teeth.
- Slide the brush in and out of the space 3-5 times using the full length of the bristle part of the brush. This action will remove the dental plaque.
- Do not push interdental brushes between the teeth with force and high speed. Slight and controlled pressure of the brush against the gums should be used, as it will allow the bristles to penetrate slightly underneath the gum margin.
- By slightly closing the mouth, it will be easier to manipulate the brush as the tension in the cheeks is lessened. It might also be helpful to bend the brush slightly to ease insertion.
- Cleanse all areas between the teeth where an interdental brush will fit. Rinse interdental brushes thoroughly after use and allow them to dry out.
- It is often a good idea to combine the use of interdental brushes and woodsticks or non-wire interdental cleaning devices.
- After use, carefully rinse and store the interdental brush so that it can dry in the air to reduce bacterial growth.

For the best individualized approach, it is advisable to seek the advice of your dental care professional.

*The instructions provided here are adapted and updated. The original instructions are sourced from "Mechanical Supragingival Plaque Control" by F. van der Weijden and D. E. Slot, as found in Lindhe’s Clinical Periodontology and Implant Dentistry, Chapter 28, 7th Edition T. Berglundh, with W. V. Giannobile, N. P. Lang, and M. Sanz.

**Appendix S3.** Dental floss implementation: recommendations for optimal usage and oral health benefits

**Dental floss implementation: recommendations for optimal usage and oral health benefits**

Dental floss and tape, with tape being a broader type, are most useful when interdental papillae completely fill the embrasure space. Various floss types, like waxed and unwaxed, exist, with studies showing no difference in their effectiveness. Unwaxed floss is recommended for normal tooth contacts due to its easy sliding and thinness. It covers a larger tooth surface when it separates during use. Waxed floss suits tight proximal contacts. Step-by-step instructions and regular reinforcement are vital for effective flossing. The objective of flossing is to remove plaque, not just food particles.

**Instructions*:**

- Begin with approximately 40 cm of dental floss, loosely winding the ends around your middle fingers, leaving a 10 cm gap between them. Hold the floss taut with about 3 cm between your thumbs or create a loop.
- Employ a gentle sawing motion as you guide the taut floss between front and back teeth, taking care in tight spaces to avoid snapping that could harm the gums.
- Form a "U" shape around one tooth, pressing firmly against the side, and carefully slide the floss just under the gum using an up and down motion.
- Draw the floss up to the proximal contact, then repeat the process on the adjacent tooth bordering the gum-filled space.
- Safely remove the floss with a sawing motion and repeat the entire process for all other spaces in the mouth.
- Ensure hygiene by using a fresh section of floss for each space, unwinding from one middle finger while winding it around the other.

This process should not be painful but don't be concerned if your gums bleed initially; this should subside with continued flossing. Persistence is key, so don't give up!

For the best individualized approach, it is advisable to seek the advice of your dental care professional.

*The instructions provided here are adapted and updated. The original instructions are sourced from "Mechanical Supragingival Plaque Control" by F. van der Weijden and D. E. Slot, as found in Lindhe’s Clinical Periodontology and Implant Dentistry, Chapter 28, 7th Edition T. Berglundh, with W. V. Giannobile, N. P. Lang, and M. Sanz.

**Appendix S4.** Non wire Interdental Cleaning Devices implementation: recommendations for optimal usage and oral health benefits

**Non wire Interdental Cleaning Devices implementation: recommendations for optimal usage and oral health benefits**

Many adults possess sufficient interdental space for the use of rubber/elastomeric interdental cleaning sticks. These sticks feature a firm yet flexible conical plastic core, either coated with a soft rubber/elastomeric layer and bristles or a flexible silicone covering and lamellae. While resembling interdental brushes, their working effect is akin to woodsticks. They come in various sizes, and selecting the right size is crucial for optimal results. Designed for single-use, they are convenient for on-the-go use.

**Instructions*:**

- Detach an interdental cleaning stick from the strip.
- Hold the stick at the grip between the thumb and first finger, with additional fingers providing support on the chin when possible.
- Insert the stick's point into the interdental space.
- Push the stick as far into the space as possible, then pull it back slightly. Repeat this motion a few times with a light, sawing movement.
- Maintain a straight insertion angle into the interdental space.
- Apply light pressure against the gums simultaneously.
- Avoid forcing the stick into tight spaces between teeth.
- When cleaning between premolars and molars, slightly close the mouth to reduce cheek tension, facilitating movements.
- Attempt to clean all interdental spaces with one stick, but if it bends, use a new one.
- After use, dispose of the used interdental cleaner stick in the trash basket.

*The instructions provided here are adapted and updated. The original instructions are sourced from "Mechanical Supragingival Plaque Control" by F. van der Weijden and D. E. Slot, as found in Lindhe’s Clinical Periodontology and Implant Dentistry, Chapter 28, 7th Edition T. Berglundh, with W. V. Giannobile, N. P. Lang, and M. Sanz.

**Appendix S5.** Woodsticks implementation: recommendations for optimal usage and oral health benefits

**Woodsticks implementation: recommendations for optimal usage and oral health benefits**

Wood sticks are available in various thicknesses, are triangular and designed to match the interdental space. They are single-use and convenient for quick moments.

**Instructions*:**

- Hold the stick midway with a firm grip between the thumb and first finger. Use other fingers for support on the chin when possible. Soften the tip by moistening it through gentle sucking.
- Place the flat side of the stick against the gum, ensuring the flat surface faces upward in the upper jaw and downward in the lower jaw.
- Push the stick firmly from the outer side into the space until wedged. Pull it back slightly and push again using a light, sawing motion perpendicular to the outer teeth surfaces. Apply light pressure to the gums simultaneously. Repeat, angling the stick to contact tooth surfaces enclosing the space.
- When using between premolars and molars, slightly close the mouth to ease movements by reducing cheek tension.
- Clean all spaces between teeth with this method. If the stick pricks the gum, adjust the angle accordingly.

While in use, the device may start to disintegrate. Once you notice the initial indications of disintegration, it is advisable to dispose of the device.

If gums bleed initially, continue use; bleeding typically stops after repeated stick use. Persevere and do not give up. However, the use of sticks should not create pain.

*The instructions provided here are adapted and updated. The original instructions are sourced from "Mechanical Supragingival Plaque Control" by F. van der Weijden and D. E. Slot, as found in Lindhe’s Clinical Periodontology and Implant Dentistry, Chapter 28, 7th Edition T. Berglundh, with W. V. Giannobile, N. P. Lang, and M. Sanz.

**Appendix S6.** Oral Irrigator: recommendations for optimal usage and oral health benefits

**Oral Irrigator implementation: recommendations for optimal usage and oral health benefits**

Before utilizing any oral irrigator, it is important to thoroughly review the manufacturer's instructions, ensuring a clear understanding of its operation. Familiarizing oneself with the device's functionality and proper usage guidelines is paramount to effective and safe oral hygiene practices

**Instructions*:**

- Fill the water reservoir with lukewarm water and plug the power cord into the wall outlet. You can use a cup to fill the reservoir. If the unit has removable tips, press the appropriate tip firmly into the irrigator handle. The tip should snap into place because it works under pressure and may shoot away otherwise.
- Test the oral irrigator before use.
- Breathe calmly through your nose. Lean over the sink, and close your lips enough to prevent splashing, while still allowing water to fall from the mouth into the sink.
- Aim the tip just above and toward the gum line at a 90 degrees angle, and press the switch that allows the water to flow.
- Do not attempt to watch yourself in the mirror. You will make a mess!
- Starting with the back teeth (where your molars are located), follow the gum line. Take your time to get in between teeth. Continue to work slowly forward until all areas around and between teeth have been cleaned.
- Use the same sequence each time you use the irrigator so that you do not miss any teeth.
- At difficult to reach areas you can adjust the angle of the nozzle, for example while cleaning the brackets of an orthodontic appliance or at root furrows.
- Spit out excess water as needed.
- Empty any water remaining in the reservoir after use. Dry thoroughly to avoid bacterial growth. Make sure to unplug the unit before cleaning it. Irrigating is a technique that relies on your sense of touch. At first, it might take a little longer until you develop a routine and become more comfortable with the oral irrigator. Depending on the power level, you might need to refill the water reservoir. Antiseptics can be added if that has been advised by your dental care professional. If so, a mouth rinse or another antiseptic is added to the water in the reservoir.

For the best individualized approach, it is advisable to seek the advice of your dental care professional.

*The instructions provided here are adapted and updated. The original instructions are sourced from "Mechanical Supragingival Plaque Control" by F. van der Weijden and D. E. Slot, as found in Lindhe’s Clinical Periodontology and Implant Dentistry, Chapter 28, 7th Edition T. Berglundh, with W. V. Giannobile, N. P. Lang, and M. Sanz.
